# Post‐progression survival is highly linked to overall survival in patients with non‐small‐cell lung cancer harboring sensitive *EGFR* mutations treated with first‐line epidermal growth factor receptor‐tyrosine kinase inhibitors

**DOI:** 10.1111/1759-7714.13193

**Published:** 2019-10-08

**Authors:** Hisao Imai, Kyoichi Kaira, Keita Mori, Mie Kotake, Masumi Mitani, Naoko Kawashima, Takeshi Hisada, Koichi Minato

**Affiliations:** ^1^ Division of Respiratory Medicine Gunma Prefectural Cancer Center Ota Japan; ^2^ Department of Respiratory Medicine Comprehensive Cancer Center, International Medical Center, Saitama Medical University Hidaka Japan; ^3^ Clinical Research Promotion Unit Clinical Research Center, Shizuoka Cancer Center Nagaizumi Japan; ^4^ Division of Pharmacy Gunma Prefectural Cancer Center Ota Japan; ^5^ Gunma University Graduate School of Health Sciences Maebashi Japan

**Keywords:** Advanced non‐small‐cell lung cancer, *EGFR* mutations, EGFR‐TKIs, introduction, overall survival, post‐progression survival

## Abstract

**Background:**

In patients with epidermal growth factor receptor (*EGFR*)‐mutated advanced non‐small‐cell lung cancer (NSCLC), epidermal growth factor receptor‐tyrosine kinase inhibitor (EGFR‐TKI) treatment has shown a good response. Subsequent treatments jeopardize the ability to determine the effect of first‐line chemotherapy on overall survival (OS). Therefore, using patient‐level data, we aimed to study the associations of progression‐free survival (PFS) and post‐progression survival (PPS) with OS after first‐line EGFR‐TKI treatment in patients with *EGFR*‐mutated NSCLC.

**Methods:**

Between November 2006 and December 2016, we analyzed 92 patients with *EGFR*‐mutated NSCLC treated with first‐line EGFR‐TKI. The correlations of PFS and PPS with OS were analyzed for each patient.

**Results:**

Spearman's rank correlation and linear regression analyzes showed that PPS correlated highly with OS (*r* = 0.85, *P* < 0.05, *R* ^*2*^ = 0.75), whereas PFS correlated weakly with OS (*r* = 0.76, *P* < 0.05, *R* ^*2*^ = 0.50). The best responses after first‐line and second‐line treatments were significantly associated with PPS.

**Conclusions:**

PPS has a higher impact on OS than PFS in patients with *EGFR*‐mutated NSCLC treated with first‐line EGFR‐TKIs. These outcomes suggest that the OS in this patient group may be affected by treatments following first‐line chemotherapy; however, this remains to be verified in prospective trials.

## Introduction

Lung cancer is the most common cause of cancer‐related deaths worldwide, with non‐small‐cell lung cancer (NSCLC) accounting for ~85% of all lung cancer cases.^1^ Most patients with NSCLC are diagnosed at the advanced stage of the disease, which is associated with notably poor outcomes. Previous clinical trials have shown the effectiveness of epidermal growth factor receptor‐tyrosine kinase inhibitors (EGFR‐TKIs) such as gefitinib, erlotinib, and afatinib, as a first‐line treatment for patients with *EGFR*‐mutated NSCLC.^2^, ^3^, ^4^, ^5^, ^6^, ^7^

With the increase in the number of pharmacological agents and combination regimens for NSCLC chemotherapy, the effect of first‐line chemotherapy on overall survival (OS) may be jeopardized by subsequent treatments.^8^ In a recent randomized study on NSCLC patients, a favorable shift in progression‐free survival (PFS) did not necessarily improve OS.^9^ However, PFS following first‐line chemotherapy is not a valid substitutional endpoint for OS. Alternatively, over the last decade, post‐progression survival (PPS) has been shown to be strongly associated with OS after first‐line chemotherapy for advanced NSCLC with drugs against molecular targets, such as gefitinib and erlotinib, which remedied advanced NSCLC.^10^, ^11^ PPS is calculated as the difference between OS and PFS.^12^ A previous report showed that unlike PFS and tumor shrinkage, PPS was significantly associated with OS after the initiation of second‐line treatment, suggesting that any further treatment after disease progression following first‐line treatment may significantly affect OS in patients with advanced *EGFR*‐mutated NSCLC treated with gefitinib.^13^

EGFR‐TKIs are the standard first‐line treatments for *EGFR*‐mutated NSCLC. Furthermore, several treatment options in addition to first‐line EGFR‐TKI are available including platinum‐based combination chemotherapy, nonplatinum‐based chemotherapy, or other approved EGFR‐TKIs, such as osimertinib. The median survival of patients with *EGFR*‐mutated NSCLC who undergo second‐line treatment with EGFR‐TKIs, platinum‐based combination chemotherapy, and pemetrexed or docetaxel is approximately three years.^14^ Approximately 60% of patients (irrespective of race or ethnic background) who relapsed after first‐line EGFR‐TKI treatment harbored a T790M mutation in EGFR.^15^, ^16^, ^17^, ^18^ Osimertinib is the standard second‐line treatment in patients with advanced progressive T790M‐positive NSCLC after treatment relapse with first‐line EGFR‐TKI treatment.^19^ In addition, patients with advanced T790M‐negative NSCLC after treatment relapse with first‐line EGFR‐TKI treatment are managed with cytotoxic drug chemotherapy.

Results on survival after administration of therapies post disease progression are interesting at the patient‐level. Previous assessments of individual data implied that PPS correlates strongly with OS after first‐line treatment in patients with advanced NSCLC and small‐cell lung cancer (SCLC), whereas PFS correlates weakly with OS.^20^ Therefore, subsequent treatment due to disease progression following first‐line chemotherapy may considerably affect OS. However, whether this is veridical in patients with advanced NSCLC harboring *EGFR* mutations sensitive to molecule‐targeted treatment is unclear. Therefore, the examination of patient‐level data to determine whether PFS and PPS correlate significantly with OS following first‐line treatment in these patients will be clinically fruitful.

In this study, we assessed the associations of PFS and PPS with OS in patients with advanced or metastatic *EGFR*‐mutated NSCLC. We also evaluated the prognostic usability of patient characteristics for PPS.

## Methods

### Patients

**Figure 1:**
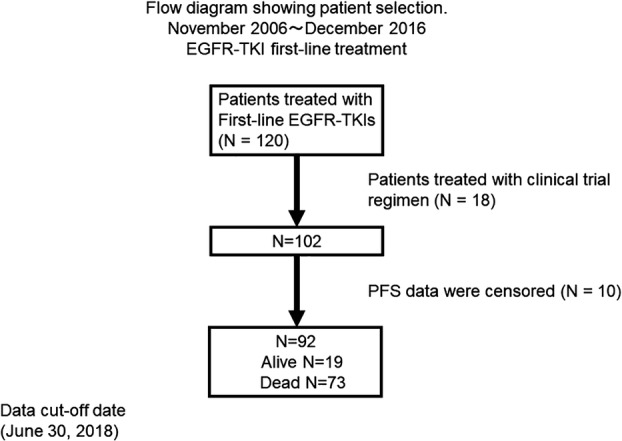
Flow chart showing patient selection. The patients received epidermal growth factor receptor‐tyrosine kinase inhibitor first‐line chemotherapy between November 2006 and December 2016. PFS, progression‐free survival.

This investigation included 120 patients with advanced *EGFR*‐mutated NSCLC who were administered first‐line EGFR‐TKIs between November 2006 and December 2016 at the Gunma Prefectural Cancer Center. The histological diagnosis and stage of NSCLC were based on the World Health Organization's classification and the American Joint Committee on Cancer's tumor‐node‐metastasis (TNM) staging system,^21^ respectively. The eligibility criteria were histopathologically‐or cytologically‐proven NSCLC, unresectable stage disease, tumor with a drug‐sensitive *EGFR* mutation (exon 18 G719X, exon 19 deletion, exon 21 L858R, or exon 21 L861Q), and disease progression beyond first‐line EGFR‐TKI treatment. All patients were EGFR‐TKI naïve, initially treated with gefitinib (250 mg/day), erlotinib (150 mg/day), or afatinib (30 or 40 mg/day), except first‐line third‐generation EGFR‐TKIs such as osimertinib as third‐generation EGFR‐TKIs were not approved for first‐line treatment during the study period, and then confirmed to have progressive disease. Prior to the treatment, each patient underwent physical examination, chest radiography, thoracic and abdominal computed tomography, bone scintigraphy or ^18^F‐fluorodeoxyglucose positron emission tomography, and brain computed tomography or magnetic resonance imaging to evaluate the TNM stage. The medical records of the identified and selected patients were reviewed at a hospital. Data on baseline characteristics, chemotherapy regimens, responses to first‐line EGFR‐TKI treatment, and whether second‐line and subsequent‐line chemotherapy were administered were obtained. The second‐line and subsequent‐line regimens were decided by the attending physician and were continued until disease progression, unacceptable adverse events, or withdrawal of agreement. After relapse following first‐line EGFR‐TKI treatment, patients were permitted to select any subsequent mode of treatment after the administration of EGFR‐TKIs. A total of 18 patients were treated with clinical trial regimens of EGFR‐TKI plus cytotoxic drugs or combination chemotherapy with other molecule‐targeted drugs, and the PFS data for 10 patients were censored. To ensure a uniform patient background, these 28 patients were excluded from the analysis. Thus, 92 patients were retrospectively analyzed (Fig 1).

Sensitive *EGFR* mutations in exons 18–21 were analyzed as previously described.^22^, ^23^ The sensitive *EGFR* mutations were determined using polymerase chain reaction (PCR) amplification and intron‐exon boundary primers. In this study, exon 18 G719X, exon 19 deletion, exon 21 L858R, and exon 21 L861Q were considered sensitive *EGFR* mutations. Exon 19 deletion and exon 21 L858R were major sensitive mutations, whereas others were minor sensitive mutations.

The study protocol was approved by the Institutional Review Board of the Gunma Prefectural Cancer Center. The requirement for written informed consent was waived owing to the retrospective nature of the study.

### Response evaluation

The best overall response and maximum tumor shrinkage were recorded as tumor responses. Radiographic tumor responses were defined according to the Response Evaluation Criteria in Solid Tumors, version 1.1.^24^ Complete response (CR) was defined as the disappearance of all target lesions; partial response (PR) was characterized by a decrease in the sum of the diameters of the target lesion by at least 30% compared to baseline; progressive disease (PD) was associated with an increase of at least 20% in the sum of the diameters of the target lesion compared to the smallest sum during the study; stable disease (SD) was characterized by insufficient shrinkage or expansion to qualify as PR or PD.

### Statistical analysis

PFS was measured from the initiation of treatment until PD or death due to any reason, and OS was measured from the first day of treatment until death or the date of the last follow‐up. PPS was recorded as the time from tumor progression until death or the date of the last follow‐up. The survival curves were calculated using the Kaplan‐Meier method. Linear regression analysis was used to evaluate whether PFS and PPS correlated with OS. To explore the prognostic factors for PPS, we used the proportional hazards model with a stepwise regression procedure. Hazard ratios (HRs) and 95% confidence intervals (CIs) were evaluated. As HR is defined for a one unit difference, certain factors were converted to an appropriate measure. PPS values were compared using the log‐rank test. *P* < 0.05 was considered statistically significant. The two‐tailed significance level was set at 0.05. All statistical analyzes were conducted using JMP, version 11.0, for Windows (SAS Institute, Cary, NC, USA).

## Results

### Patient characteristics and therapeutic efficiency

Of the 92 patients included in the current study, 73 died due to their primary disease. The median follow‐up time was 18.3 months (range, 0.5–75.3 months). The patients' median age was 69 years (range, 39–87 years). The patient characteristics are shown in Table 1.

**Table 1:** Baseline patient characteristics

Characteristics	*N* = 92
Gender	
Male/Female	35 / 57
Median age at treatment (years)	69 (39–87)
Performance status (PS)	
0 / 1 / 2/ ≧3	37 / 38 / 8 / 9
Smoking history	
Yes / No / unknown	37 / 55 / 0
Histology	
Adenocarcinoma/others	91 / 1
Clinical stage at diagnosis	
II / III / IV / postoperative recurrence	1 / 4 / 69 / 18
First‐line EGFR‐TKI	
Gefitinib / erlotinib / afatinib	74 / 11 / 7
Mutation type	
exon 19 del / exon 21 L858R / others	41 / 46 / 5
Presence of T790 mutation at recurrence	
Yes / No / unknown	10 / 7 / 75
Administration of first or second generation EGFR‐TKI rechallenge	
Yes / No	24 / 68
Administration of osimertinib	
Yes / No	8 / 84
Administration of immune checkpoint inhibitors	
Yes / No	5 / 87
Number of regimens after progression	
0 / 1 / 2 / 3 / 4 / 5 / ≧6	39 / 28 / 14 / 6 / 2 / 1 / 2
Median (range)	1 (0–6)

EGFR‐TKI, epidermal growth factor receptor‐tyrosine kinase inhibitor.

**Figure 2:**
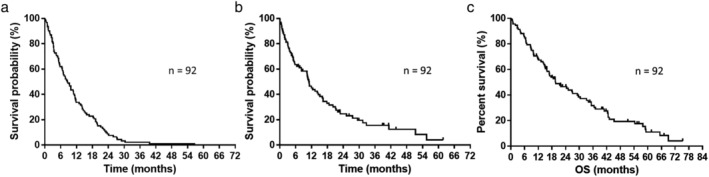
(**a**) Kaplan‐Meier plots showing progression‐free survival (PFS). Median progression‐free survival: 8.6 months. (**b**) Kaplan‐Meier plots showing post‐progression survival (PPS). Median post‐progression survival: 11.0 months. (**c**) Kaplan‐Meier plots showing overall survival (OS). Median overall survival: 19.6 months.

Among the 92 patients treated with first‐line EGFR‐TKIs, 0, 61, 15, and 16 showed CR, PR, SD, and PD, respectively. The response rate was 66.3% (95% confidence interval [CI], 56.6%–75.9%), and the disease control rate was 82.6% (95% CI, 74.8%–90.3%). For disease progression after first‐line EGFR‐TKI treatment, 53 of 92 cases were administered the following treatment (including three patients who were administered second‐line treatment along with continuation of first‐line EGFR‐TKI), whereas the remaining 39 patients did not receive any chemotherapy. Among the 92 patients, the median number of regimens after disease progression beyond the first‐line EGFR‐TKI treatment was one (range: 0–6). The chemotherapeutic regimens administered beyond disease progression are listed in Table 2. Platinum combination chemotherapeutic regimens were the most common second‐line treatment. The median PFS, PPS, and OS were 8.6, 11.0, and 19.6 months, respectively (Fig 2(a)–(c)).

**Table 2:** Chemotherapy regimens used after progression following first‐line EGFR‐TKI treatment

	Second‐line	≧Third‐line	Total
Platinum combination	24	2	26
Docetaxel	3	6	9
Pemetrexed	8	3	11
S1	0	6	6
First or second generation EGFR‐TKI rechallenge	15	9	24
Osimertinib	3	5	8
Immune checkpoint inhibitors	0	5	5
Others	0	9	9
Investigational agent	0	0	0

EGFR‐TKI, epidermal growth factor receptor‐tyrosine kinase inhibitor.

### Correlation of PFS and PPS with OS

**Figure 3:**
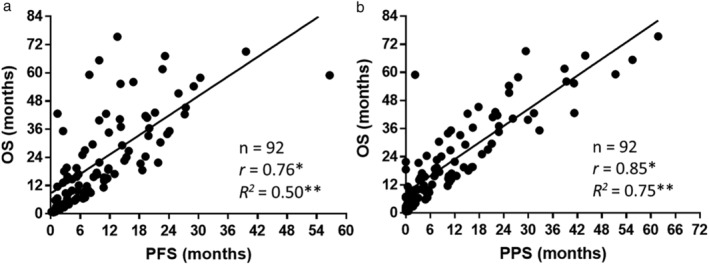
(**a**) Correlation between overall survival (OS) and progression‐free survival (PFS). (**b**) Correlation between overall survival (OS) and post‐progression survival (PPS). * *r* values represent Spearman's rank correlation coefficient, ** *R* ^*2*^ values represent linear regression.

The correlation of PFS and PPS with OS is shown in Fig 3(a) and (b). Using Spearman's rank correlation coefficients and linear regression, PPS was shown to be highly associated with OS (*r* = 0.85, *P* < 0.05, *R* ^*2*^ = 0.75), unlike PFS (*r* = 0.76, *P* < 0.05, *R* ^*2*^ = 0.50).

### Clinical factors influencing PPS

**Figure 4:**
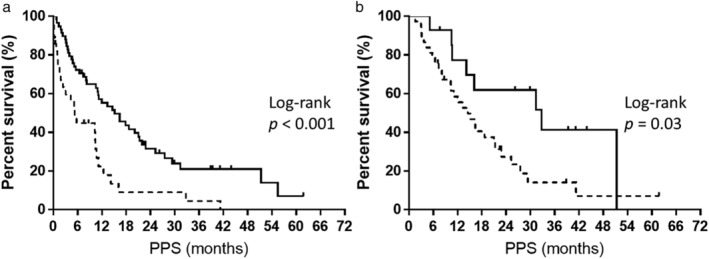
(**a**) Kaplan‐Meier plots showing post‐progression survival (PPS) according to best response at first‐line treatment. Partial response (PR), median: 15.1 months; nonpartial response (Non‐PR), median: 5.2 months. (**b**) Kaplan‐Meier plots showing post‐progression survival (PPS) according to best response at second‐line treatment. Partial response (PR), median: 32.7 months; nonpartial response (non‐PR), median: 14.5 months. 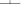 PR, 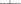 Non‐PR.

Univariate analysis revealed performance status (PS) at the beginning and end of first‐line treatment, the best response at first‐and second‐line treatment with or without administration of EGFR‐TKI rechallenge, osimertinib, or immune checkpoint inhibitors, and the number of regimens after disease progression following first‐line EGFR‐TKI treatment, which were the factors that correlated with PPS (*P* < 0.05) (Table 3). Multivariate analysis indicated that the best responses at first‐line and second‐line treatment (PR vs. non‐PR) were the clinical factors influencing PPS (*P* < 0.05, Table 4). Log‐rank tests were used to corroborate that these two factors significantly correlated with PPS (log‐rank test, *P* < 0.05; 4(a) and (b)). Cases of PR at first‐line treatment had a median PPS of 15.1 months; on the other hand, their non‐PR counterparts had a median PPS of 5.2 months (log‐rank test, *P* < 0.001; Fig 4(a)). Furthermore, cases with PR at second‐line treatment had a median PPS of 32.7 months, whereas their non‐PR counterparts had a median PPS of 14.5 months (log‐rank test, *P* = 0.03; Fig 4(b)). These outcomes remained congruent after adjusting for confounding factors in the Cox proportional hazards model (Table 4).

**Table 3:** Univariate Cox regression analysis of patient characteristics for post‐progression survival

	Post‐progression survival		
Factors	Hazard ratio	95% CI	*P‐*value
Gender (Male/Female	0.98	0.60–1.57	0.95
Age at the beginning of first‐line treatment	1.02	0.99–1.04	0.05
Age at the beginning of first‐line treatment (<75/≧75)	1.00	0.61–1.69	0.97
PS at the beginning of first‐line treatment	2.01	1.56–2.57	**<0.001**
Smoking status (Yes/No)	1.26	0.79–2.00	0.32
Postoperative recurrence (Yes/No)	0.75	0.39–1.34	0.35
*EGFR* mutation type			
Major mutation/minor mutation	0.72	0.29–2.38	0.55
First‐line EGFR‐TKI, firstfirst generation (gefitinib/erlotinib)/2nd generation (afatinib)	1.71	0.63–7.01	0.32
Best response at first‐line treatment			
PR/non‐PR	0.39	0.24–0.65	**<0.001**
Non‐PD/PD	0.46	0.25–0.90	**0.02**
PS at the end of first‐line treatment	2.79	2.11–3.71	**<0.001**
Age at the beginning of second‐line treatment	1.01	0.98–1.05	0.29
PS at the beginning of second‐line treatment	1.66	0.91–3.06	0.09
Best response at second‐line treatment			
PR/non‐PR	0.42	0.17–0.90	**0.02**
Non‐PD/PD	0.30	0.14–0.63	**0.002**
Administration of EGFR‐TKI rechallenge (Yes/No)	0.59	0.33–1.01	0.05
Administration of osimertinib (Yes/No)	0.36	0.11–0.89	**0.02**
Administration of immune checkpoint inhibitors (Yes/No)	0.36	0.08–0.99	**0.04**
Number of regimens after progression beyond first‐line EGFR‐TKI treatment	0.61	0.47–0.76	**<0.001**

Values in bold type are significant (*P* **<** 0.05). CI, confidence interval; *EGFR*, epidermal growth factor receptor disease gene; EGFR‐TKI, epidermal growth factor receptor‐tyrosine kinase inhibito; PD, progressiver; PR, partial response; PS, performance status.

**Table 4:** Multivariate Cox regression analysis of factors correlated with PPS

	Post‐progression survival		
Factors	Hazard ratio	95% CI	*P‐*value
PS at the beginning of first‐line treatment	1.70	0.92–3.02	0.08
Best response at first‐line treatment			
PR/non‐PR	0.45	0.20–0.99	**0.04**
PS at the end of first‐line treatment	1.08	0.54–2.16	0.81
Best response at second‐line treatment			
PR/non‐PR	0.33	0.13–0.75	**0.007**
Administration of osimertinib (Yes/No)	1.14	0.31–3.25	0.81
Administration of immune checkpoint inhibitors (Yes/No)	0.56	0.11–1.93	0.38
Number of regimens after progression beyond first‐line EGFR‐TKI treatment	0.94	0.65–1.29	0.72

Values in bold type are significant (*P* **<** 0.05). CI, confidence interval; EGFR‐TKI, epidermal growth factor receptor‐tyrosine kinase inhibitor; PD, progressive disease; PR, partial response; PS, performance status.

### Long‐term survivors

**Figure 5:**
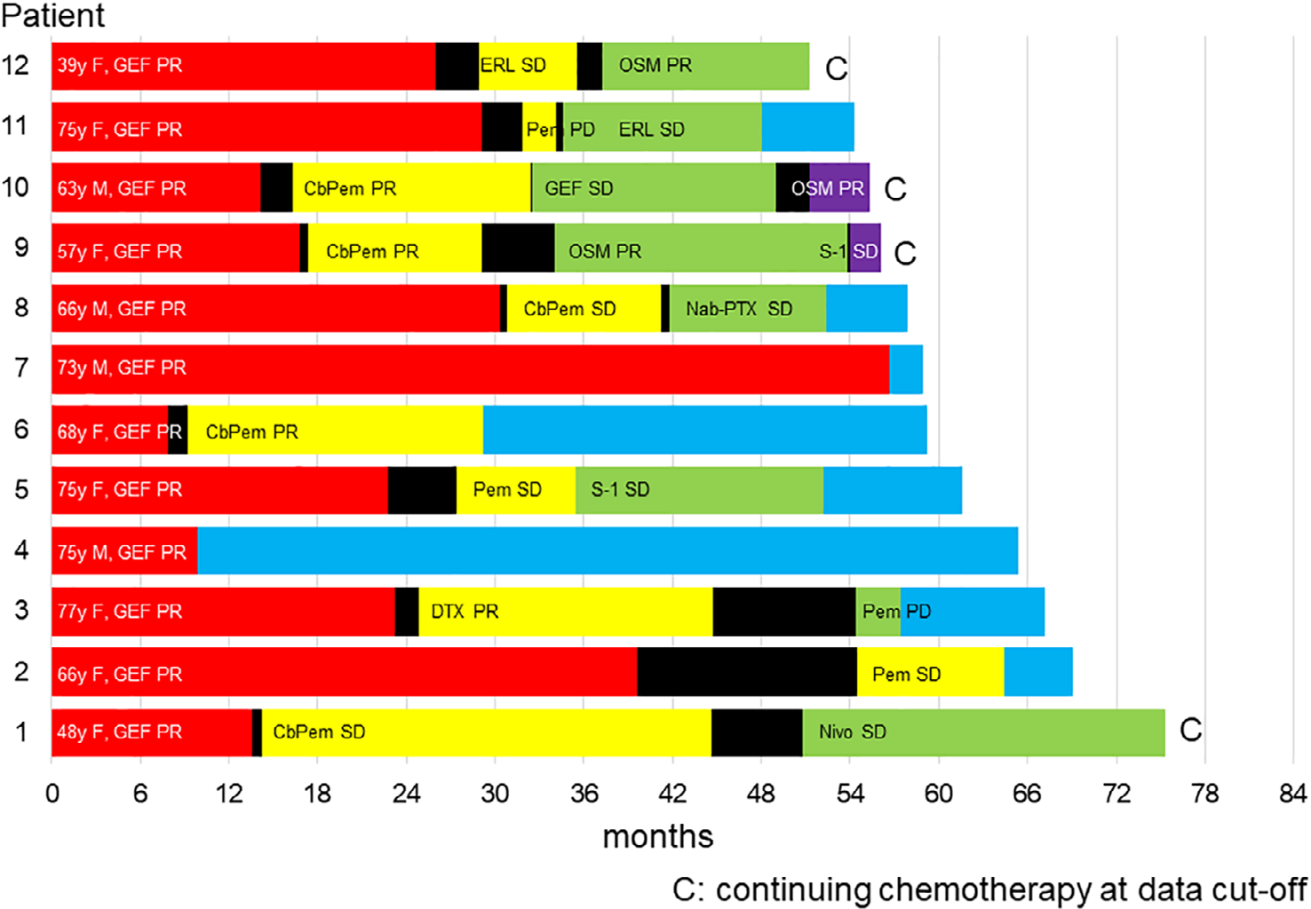
Details of treatment courses in patients who survived for more than four years. CbPem, carboplatin plus pemetrexed; DTX, docetaxel; EGFR‐TKI, epidermal growth factor receptor‐tyrosine kinase inhibitor; ERL, erlotinib; F, female; GEF, gefitinib; M, male; Nab‐PTX, Nab‐paclitaxel; Nivo, nivolumab; OSM, osimertinib; PD, progressive disease; Pem, pemetrexed; PR, partial response; SD, stable disease. 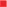 1^st^‐line EGFR‐TKIs, 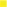 2^nd^ line treatment, 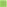 3^rd^ line treatment, 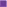 4^th^ line treatment, 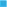 Best supportive care, 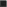 Treatment free interval.

A total of 12 patients survived for more than four years. Figure 5 shows the individual characteristics and survival status of these patients. Patient characteristics with respect to PS, clinical stage, and histology were as follows: all 12 patients had PS 0, one patient had stage IIIB disease, eight patients had stage IV disease, three patients showed postoperative recurrence, and all 12 patients had adenocarcinoma. All patients were administered gefitinib as the first‐line EGFR‐TKI treatment and exhibited PR. The median PFS of first‐line EGFR‐TKI treatment was 22.9 months in all 12 patients. Ten of twelve patients were administered EGFR‐TKI for more than one year. Three patients were administered only EGFR‐TKIs. The others were also treated with cytotoxic chemotherapy. Four patients continued chemotherapy at data cutoff. Among the 12 patients who survived for more than four years, two, two, six, and two patients received first‐, second‐, third‐, and fourth‐line chemotherapy, respectively.

## Discussion

We analyzed the correlation of OS with PFS and PPS at the patient‐level in cases with *EGFR*‐mutated NSCLC treated using first‐line EGFR‐TKIs. PPS showed more significant correlation with OS than PFS. In addition, PPS was affected by the best response at first‐line and second‐line treatment (PR vs. non‐PR).

The efficacy of substitutional endpoints has been previously evaluated using meta‐analysis.^25^, ^26^ Recently, experts of biological statistics have proposed various methodologies for certifying surrogate endpoints.^27^, ^28^ Although PFS is a potent surrogate endpoint for OS in extensive diseases such as SCLC,^29^, ^30^ its utility remains controversial. Broglio *et al*. recently focused on PPS in a hypothetical clinical study situation under the hypothesis that therapy influenced PFS but not PPS.^12^ A modern‐era clinical study suggested that PPS was highly associated with OS after first‐line treatment for advanced NSCLC,^10^, ^11^ and previously we have reported the effect of PPS on advanced NSCLC and SCLC based on individual patient investigation.^31^, ^32^, ^33^, ^34^, ^35^, ^36^

In contrast to the results from previous studies,^29^, ^30^ we did not observe PFS to be a surrogate endpoint for OS in our patients with *EGFR*‐mutated NSCLC administered first‐line EGFR‐TKI, although PPS was not evaluated in the previous reports. We investigated effects pertaining to first‐line treatment, which showed that PFS did not affect OS in such situations. We observed that PFS was shorter than PPS; thus, PPS, rather than PFS, is more strongly associated with OS, and the correlation was linear (Fig 3(a) and (b)). PPS was composed of a large proportion of OS, implying that treatment was weak for PFS to extend OS. Therefore, in clinical trials where patients are expected to have a brief PFS after first‐line treatment, factors that affect PPS will predominate similar to that observed in our analysis, and we must control for factors that affect the PPS.

A previous clinical study for advanced NSCLC demonstrated that an extended PPS in first‐line monotherapy and a molecule targeting agent were associated with good PS.^10^ However, the factors affecting PPS in individual cases with *EGFR*‐mutated NSCLC treated with first‐line EGFR‐TKI are obscure. Log‐rank tests identified that two factors were strongly associated with and were prognostic for PPS, namely, best response at first‐line and second‐line treatments (PR *vs*. non‐PR). Depths of response achieved by the first‐line EGFR‐TKI treatment could affect PPS. This may be due to the significant reduction in tumor size obtained with first‐line EGFR‐TKI treatment. These observations indicated that response to first‐line and second‐line treatments in patients with *EGFR*‐mutated NSCLC might be useful for extending PPS and consequently OS. The large number of drugs used after disease progression is possibly the result of the increase in the number of active agents currently available for second‐ and third‐line chemotherapy for advanced NSCLC, such as platinum combination chemotherapy, docetaxel, pemetrexed, S1, osimertinib, and immune checkpoint inhibitors (Table 2). Similarly, monotherapy with new‐generation EGFR‐TKIs with more specific activity for the T790M mutation, such as osimertinib, show better adverse event profiles in clinical trials, and the outcomes are encouraging in patients with advanced NSCLC who develop a secondary T790M mutation and EGFR‐TKI resistance.^15^ Rebiopsy might be valuable in decision‐making for follow‐up treatment. However, most patients in our current study died before the test for T790M was established; thus, the T790M status of most patients was not examined. Osimertinib has a significant impact on the treatment for advanced NSCLC harboring sensitive *EGFR* mutations. If many patients who progress to a secondary T790M mutation are managed with osimertinib, the latter might have a more significant impact on the PPS than the current scenario. Furthermore, osimertinib administration was associated with longer PFS than current standard first‐line treatments for *EGFR*‐mutated NSCLC.^37^ Hence, osimertinib might be a more effective standard first‐line treatment for *EGFR* mutation‐positive NSCLC. As second‐line chemotherapy is currently changing, the PPS after first‐line treatment in this patient group might also change. PPS will exert higher impact on the OS of patients with NSCLC harboring *EGFR* T790M mutations treated with first or second‐generation EGFR‐TKIs as the first‐line treatment when osimertinib is used as the second‐line treatment. However, PPS might also be valuable if osimertinib is used as the first‐line treatment. Despite the difficulty of following up treatments in patients with *EGFR*‐mutated NSCLC treated with first‐line EGFR‐TKIs, our observations imply that OS correlates more strongly with PPS than PFS in patients with *EGFR*‐mutated NSCLC who received EGFR‐TKIs as the first‐line of treatment. Consequently, subsequent treatments might be beneficial for extending OS in these patients. However, multivariate analysis demonstrated that administration of osimertinib and immune checkpoint inhibitors were not independent prognostic factors for PPS in our cohort. This might be due to the modest sample size, which limited our ability to evaluate the relationship between PPS, administration of osimertinib, and immune checkpoint inhibitors.

Among long‐term survivors (survived more than four years), all patients showed PS 0 and adenocarcinoma. The majority of patients received cytotoxic drug chemotherapy as the second‐ or third‐line chemotherapy and EGFR‐TKI was administered for more than one year. Furthermore, for long‐term survivors of more than four years (patients with NSCLC harboring *EGFR* mutations), treatment with both EGFR‐TKI and cytotoxic agents might be necessary. Only one long‐term survivor of more than four years received the best supportive care after first‐line EGFR‐TKI treatment. This may be because the tumor was small and indolent at the time of diagnosis. These observations are consistent with those of previous studies; Kaira *et al*. reported that long‐term survivors of more than five years (patients with pretreated NSCLC) might require treatment with not only EGFR‐TKI but also repeated cytotoxic agents.^38^ Similarly, Huang *et al*. reported that EGFR‐TKI treatment duration of more than one year plays an important role in long‐term survival.^39^

Our study has several limitations. First, the sample size was relatively small. Only a small number of advanced NSCLC patients harboring therapy‐sensitive *EGFR* mutations who were treated with first‐line EGFR‐TKIs were available at our institution. Our sample size was also limited because of our attempt to analyze patients with similar backgrounds. However, our institution treats a relatively large number of such patients, and our clinical practices and policies are mostly unified as this is a single institution. Understanding the nature of the sources of bias in this study ensures that the results are fruitful. Future studies including a larger patient population are required. Second, we could not thoroughly assess the treatments administered after disease progression following the second‐line treatment. However, our study is meaningful as only a limited number of patients received third‐line or subsequent chemotherapy. Third, the date on which a response was recorded was decided by each physician; this might have introduced variance in the PFS and tumor response rates; however, this limitation is inherent in all retrospective studies. Fourth, we used censored survival data, which does not affect our conclusion. The PFS did not change, even when the patient did not die. In addition, PPS and OS are prolonged, and PPS correlated strongly with OS.

In conclusion, PPS has a stronger influence on OS than PFS in patients with NSCLC harboring sensitive *EGFR* mutations treated with first‐line EGFR‐TKI. Similarly, best response at first‐line and second‐line treatments (PR vs. non‐PR) are significant independent prognostic factors for PPS. These results suggest that treatments administered after first‐line EGFR‐TKI treatment affect the OS of patients with *EGFR*‐mutated NSCLC. However, more extensive multicenter prospective trials are required to validate these findings in other patient cohorts and clinical situations.
